# Regression Models Predicting the Number of Deaths from the New Coronavirus Infection

**DOI:** 10.17691/stm2020.12.2.01

**Published:** 2020

**Authors:** D.V. Melik-Huseynov, N.N. Karyakin, A.S. Blagonravova, V.I. Klimko, A.P. Bavrina, O.V. Drugova, N.V. Saperkin, O.V. Kovalishena

**Affiliations:** Deputy Governor for Social Policy, Government of the Nizhny Novgorod Region, 1 Kremlin, Nizhny Novgorod, 603082, Russia; Rector, Privolozhsky Research Medical University, 10/1 Minin and Pozharsky Square, Nizhny Novgorod, 603005, Russia; Vice-Rector for Science, Privolozhsky Research Medical University, 10/1 Minin and Pozharsky Square, Nizhny Novgorod, 603005, Russia; Chief Specialist, GC “MedInvestGroup”, 27 Alexander Solzhenitsyn St., Moscow, 109004, Russia; Associate Professor, Department of Medical Physics and Informatics, Privolozhsky Research Medical University, 10/1 Minin and Pozharsky Square, Nizhny Novgorod, 603005, Russia; Associate Professor, Department of Medical Physics and Informatics, Privolozhsky Research Medical University, 10/1 Minin and Pozharsky Square, Nizhny Novgorod, 603005, Russia; Associate Professor, Department of Epidemiology, Microbiology and Evidence-Based Medicine, Privolozhsky Research Medical University, 10/1 Minin and Pozharsky Square, Nizhny Novgorod, 603005, Russia; Professor, Head of Department of Epidemiology, Microbiology and Evidence-Based Medicine, Privolozhsky Research Medical University, 10/1 Minin and Pozharsky Square, Nizhny Novgorod, 603005, Russia

**Keywords:** coronavirus infection, COVID-19; SARS-CoV-2, prediction of infection outcome, multivariate regression model, mortality prediction.

## Abstract

Predicting the development of epidemic infection caused by the COVID-19 coronavirus is a matter of the utmost urgency for health care and effective anti-epidemic measures. Given the rapidly changing initial information and the ambiguous quality of data coming from various sources, it is important to quickly optimize the existing prognostic models by using more sophisticated algorithms.

**The aim of the study** is to test the originally developed mathematical algorithms for predicting the development of the COVID-19 epidemic process.

**Materials and Methods:**

To assess the situation in China, Italy, and the USA, we used the information from Russian- and English-language sources available in official websites. The generally accepted descriptive statistics were used; mathematical modeling was based on linear regression. Statistical data processing was performed using the IBM SPSS Statistics 24.0 and R (RStudio) 3.6.0.

**Results:**

We found significant differences not only in the incidence rate of COVID-19 in the countries in question, but also in the death rate. The risk of death associated with COVID-19 is high due to the high number of severe clinical cases of the disease reported from these countries.

Two preliminary regression models were created. The first, initial model was based on the increase in new cases of infection **—** this factor was significantly associated with the outcome; the regression coefficient was 0.02 (95% CI 0.01–0.03). In the second, expanded model, in addition to the increase in new cases, the increase in the number of severe forms of infection was also considered; the regression coefficients were 0.017 (95% CI 0.012–0.022) and 0.01 (95% CI 0.008–0.011), respectively. Adding the second variable contributed to a more accurate description of the available data by the model.

**Conclusion:**

The developed regression models for infection control and predicting the number of lethal outcomes can be successfully used under conditions of spreading diseases from the group of “new infections” when primary data received from various sourced are changing rapidly and updates of the information are continually required. In addition, our initial model can produce a preliminary assessment of the situation, and the expanded model can increase the accuracy and improve the analytic algorithm.

## Introduction

The coronavirus infection, termed COVID-19, has taken a special place in the group of “new infections” and aroused a great interest not only among the medical community, but also in the society and media. This disease has already had a devastating impact on the socio-economic situation in many countries and significantly increased the burden on their health systems. The regional epidemic situation originated in southeastern China, quickly went beyond its borders [1–3]. The extensive epidemic process of COVID-19 and the high mortality registered in most parts of the world (Western Europe, USA, the Gulf countries) in conjunction with the ongoing COVID-19 epidemics in China have compelled the WHO to define this pathology as public health emergency of international concern (January 30, 2020) and since March 11, 2020, to declare this infection to be in a pandemic status [4].

The rapid spread of COVID-19 through continents and countries with different income levels, in different conditions (medical workers, passenger ships), among different social and age groups poses a serious challenge to the healthcare systems and demands an adequate response to this threat [5, 6]. This problem cannot be solved without developing new optimized methods for predicting the further development of the situation, as well as assessing risk factors of this infection, severity, and death. In addition, reliable scientific facts regarding this infection can reduce the level of social tension, tackle misinformation in the media, and prevent panic among the general public.

The spread of the SARS-CoV-2 virus, which causes COVID-19, in Russia began on January 31, 2020 [5] after two infected Chinese citizens came to Transbaikalia and the Tyumen Region (both cases ended in recovery). In addition, several cases of infection among Russian tourists on the Diamond Princess cruise ship were documented. Over a short time, the presence of COVID-19 among Russian citizens returning from countries with confirmed coronavirus infection became evident; that was followed by cases of primary and secondary transmission [7].

We aimed to develop original models based on modern mathematical algorithms, to allow us to predict the spread, severity, and mortality of the infection.

## Materials and Methods

This is a retrospective population study. The necessary primary data was extracted from daily reports by the WHO, the National Health Commission of the People’s Republic of China, the Ministry of Health of the Russian Federation, as well as from materials published by the European Centre for Disease Prevention and Control (ECDC) and the US Center for Disease Control and Prevention (CDC). We used data from open sources available in the official websites. Relevant news releases and press releases were regularly reviewed. In the Russian Federation, at the moment, data on confirmed COVID-19 cases, on hospitalized patients with signs of pneumonia, and on people who were in contact with the infected individuals are accumulated in the “Information Record System” integrated into the Uniform State Health Information System [8]. The collected information covers the data on primary and cumulative cases of COVID-19, the increase and total number of deaths and severe forms of the infection.

**Statistical data processing** was performed using the licensed programs IBM SPSS Statistics 24.0 and R (RStudio) 3.6.0. The distribution normality was tested using the Kolmogorov–Smirnov criterion. To verify differences between the groups, the non-parametric Mann–Whitney test was used, the strength of the correlation was estimated using the Spearman coefficient, and the mode of the correlation was determined using the simple and multiple linear regressions. The models were compared using the Akaike information criterion (AIC). The results are presented as Me [IQR], where Me is the median, IQR is the interquartile range (Q1–Q3), and also as the absolute values in the arithmetic and logarithmic scales. Differences were considered statistically significant at p≤0.05. The same significance level was taken for the correlations. If necessary, a 95% confidence interval (CI) was added.

## Results and Discussion

**Figure 1:**
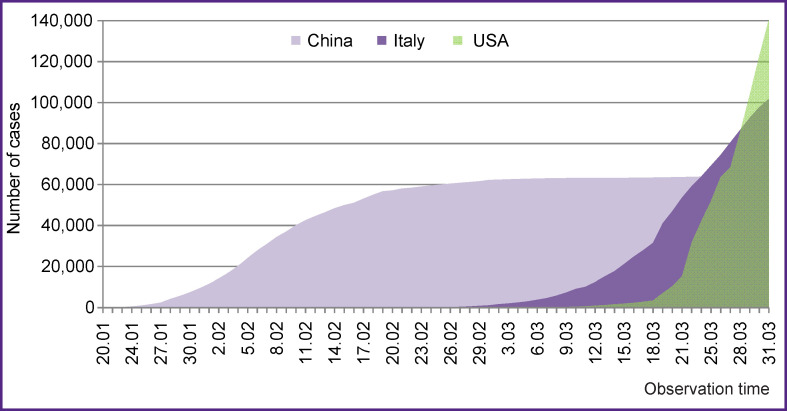
Cumulative incidence of COVID-19 in different countries (in absolute numbers) from January to March 2020

As of April 1, 2020, there were 823,626 COVID-19 cases in the world [9]. In Russia, the number of laboratory-confirmed cases of the disease amounted to 3548 (with the largest number detected in Moscow **—** 2475), of which 235 patients recovered and 30 individuals died. A total of 536,669 tests were performed all over the Russian Federation [10]. The comparison of total cases of COVID-19 (from mid-January to late March 2020) in China, Italy, and the USA showed substantial differences in the pace of the epidemic process. Thus, the incidence rates of the new coronavirus infection varied significantly across the countries (Figure 1). In China, there was a slow increase in the incidence of COVID-19, which reached a plateau 42 days after the discovery of the first case; Italy was characterized by a significantly higher incidence rate. The USA recorded a record rapid increase in the absolute number of COVID-19 cases (in particular, since March 26, 2020). At the time of the study, no plateau was reached in the latter two countries and there was a further increase in the number of cases.

**Figure 2:**
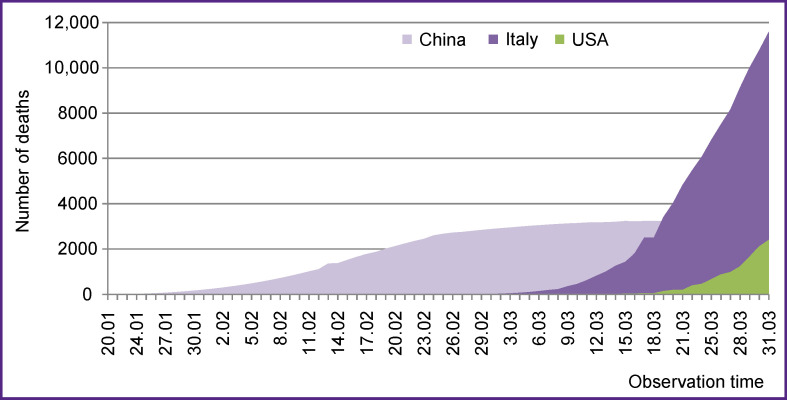
Cumulative mortality associated with SARS-CoV-2 in different countries (in absolute numbers) from January to March 2020

Mortality is an important, unambiguously assessed parameter, which is often used in epidemiological studies. Analysis of deaths associated with SARS-CoV-2 revealed the cumulative characteristics of the epidemic in each of these countries (Figure 2).

Isolated lethal cases in China have been reported since January 20, but within 1–1.5 weeks, the number of deaths increased exponentially. This increase occurred almost within one month (February), which, in all likelihood, was associated with late diagnosis and late medical care that resulted in a high number of aggravated cases. Although from early March, the number of deaths associated with COVID-19 remained high (about 3 thousand), there were no reports on subsequent growth in mortality.

Lethal outcomes in Italy showed some dramatic dynamics: over a short period, the number of deaths exceeded 1.5 thousand followed by an even steeper upward trend reaching 11,591 cases.

In the USA, the COVID-19 epidemic is characterized by a slow rise in the number of infected people, paralleled with a slow increase in the number of deaths, which amounted to 2398 cases.

**Figure 3:**
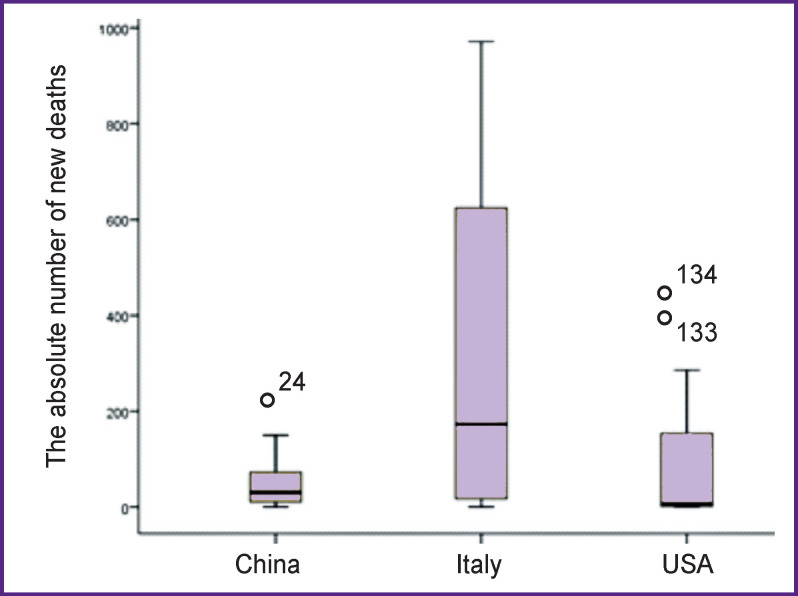
The numbers of new deaths in countries under consideration

We analyzed the incidence of coronavirus infection in this period and revealed the patterns of distribution of COVID-19 cases and new (newly recorded) deaths (Table 1, Figure 3).

**Table 1:** Descriptive statistics for newly identified cases of COVID-19

Countries	Me [IQR]*	Minimum	Maximum
China	453.00	11	3893
	[105.50–1965.75]		
Italy:			
February 23–March 7	245	48	788
March 8–March 31	4050	977	6557
USA:			
March 4–March 18	224	19	658
March 19–March 31	11,123	3355	19,332

* Using Me and IQR is inappropriate for countries that have not reached the peak incidence.

The distribution of the number of new cases of infection is characterized by significant differences between the analyzed countries (p≤0.0001). Regarding the lethal outcomes, significant differences were found only between Italy and the two other countries (p≤0.001); the differences between the USA and China did not reach statistical significance (p=0.09).

**Figure 4:**
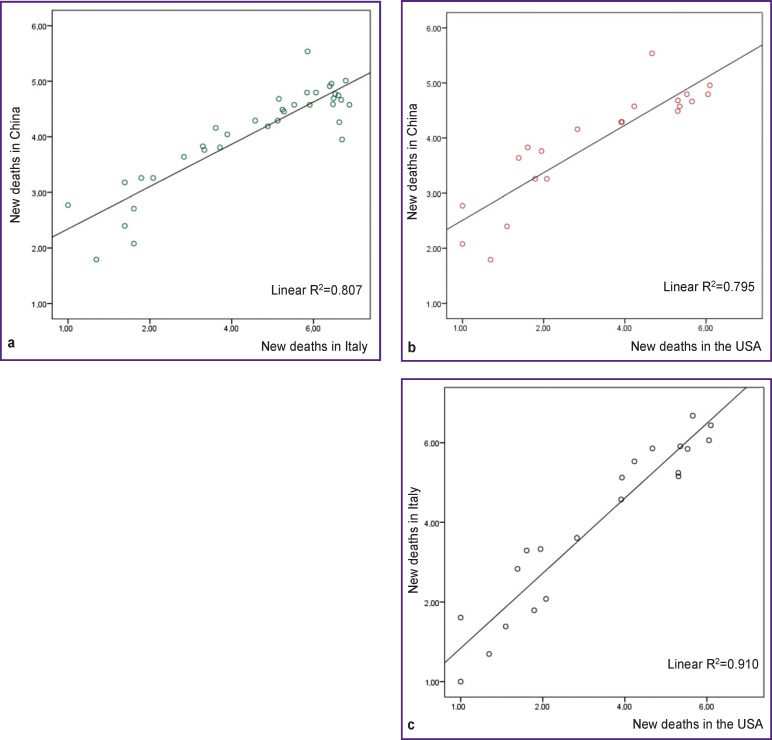
Dispersion graphs indicating a strong positive relationship between the numbers of new deaths: (a) in China and Italy (ρ=0.81; 95% CI 0.55–0.94; logarithmic scale); (b) in China and the USA (ρ=0.82; 95% CI 0.78–0.98; logarithmic scale); (c) in Italy and the USA (ρ=0.96; 95% CI 0.86–0.98; logarithmic scale); ρ is the Spearman correlation coefficient

In addition to the above differences, there was a strong positive correlation in the number of deaths between the countries (Figure 4): the correlation coefficients varied from 0.89 to 0.95 (p≤0.001).

These positive correlations suggested a unidirectional trend in the number of new deaths in all the countries studied, regardless of differences in the intensity of the epidemic process. This observation prompted us to conduct a regression analysis based on the data from China in order to develop a model that could be approximated to countries that have not reached the peak of the epidemic. Using the proposed regression equation, one can predict the number of new deaths in countries where the spread of infection is still on the rise, including in Russia.

At the early stage of the regression analysis, the initial model (version 1) was developed; in this, the regression constants β_0_ and β_1_ were determined (Table 2). This model had an AIC criterion of 709.6.

**Table 2:** The results of linear regression analysis (model 1, version 1)

Constant	Value of the constant	Standard error	Significance level	95% CI
β_0_	31.02	6.92	0.001	17.21–44.83
β_1_	0.02	0.005	0.001	0.01–0.03

The general form of the linear regression equation for this model is:

$$Y=X\cdot \beta_{1}+\beta_{0}.$$

In our study, the number of new deaths was the dependent variable *Y*, and the number of new cases of COVID-19 was the independent variable *X.* Thus, the resulting model 1 (version 1) based on this equation has the following form:

Y=X·0.02+31.02.

It is important that the obtained regression model is characterized by a determination coefficient *R*=0.5, which indicates a correct description of the source data by the model.

The next stage of the regression analysis was the creation of a predictive model, in which the variables underwent a logarithmic transformation (model 1, version 2). This form ensures the linear character of the associations. The data for building the model are shown in Table 3.

**Table 3:** The results of linear regression analysis (model 1, version 2)

Constant	Value of the constant	Standard error	Significance level	95% CI
β_0_	0.86	0.36	0.021	0.14–1.59
β_1_	0.44	0.06	0.0001	0.32–0.56

In this case, the general form of the linear regression formula for model 1 (version 2) will take the following form:

$$\text{ln}\left(Y\right)=\text{ln}\left(X\right)\cdot \beta_{1}+\beta_{0}.$$

After substituting the coefficients, we have:

ln(Y)=ln(X)·0.44+0.86.

After the logarithmic transformation was done, the determination coefficient *R* for model 1 (version 2) became 0.7, indicating an improvement in its predictive ability.

**Figure 5:**
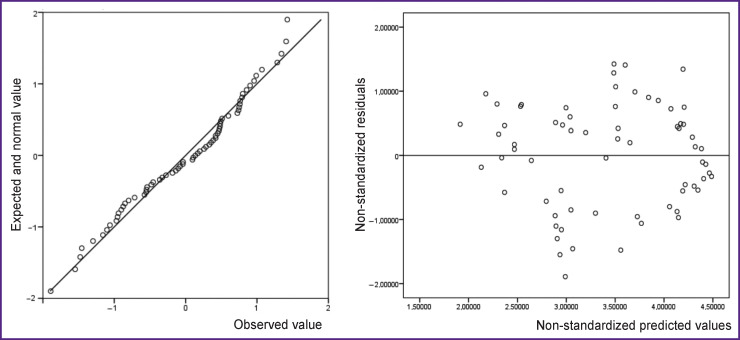
The quartile plot (Q–Q-plot) for the regression residuals and the distribution of predicted values depending on residuals (for model 1, version 2)

During the analysis, a linear association between the independent and dependent variables was tested. The condition of linearity was met as illustrated by the graphs showing the distribution of non-standardized residues (Figure 5).

These graphs reflect the uniform distribution of the numerical data, where the residue variance does not significantly change with an increase in the predicted value. This result confirms that the criteria of linearity have been met in this regression model.

Validation of the obtained models showed that predicting new lethal outcomes with high accuracy became possible after a period of 14 days when over 50 new cases of COVID-19 were registered daily. For example, when 894 patients with coronavirus infection were identified, 49 new lethal outcomes could be expected within 3–6 days after the diagnosis. When *X* goes to zero, the equation indicates a decline in the spread of infection; that same moment, the curve of the total mortality turns to a plateau. For some time, the number of new lethal cases will remain at a level equal to β_0_ (31 deaths), and then will gradually approach zero.

After we received information about the increase in the daily number of severe cases of COVID-19 in China, we decided to expand the model by adding one more factor. The proportion of severe infections is considered an important risk factor of death. In the current epidemiological situation, the Chinese health service chose to register primary cases, although the quality of the data can be debated.

The multiple regression model (model 2) was built from the data shown in Table 4. This model had an AIC criterion of 625.78.

**Table 4:** The results of multiple regression analysis (model 2)

Constant	of the Value constant	Standard error	Significance level	95% CI
β_0_	–13.31	5.1	0.008	–24.1…–3.7
β_1_	0.017	0.003	0.0001	0.012–0.022
β_2_	0.01	0.001	0.0001	0.008–0.011

The general form of the equation of multiple linear regression (model 2) is:

$$Y=\beta_{0}+X_{1}\cdot \beta_{1}+X_{2}\cdot \beta_{2},$$

where *X*_1_ is the number of infected persons; *X*_2_ — the number of severe cases; *Y* is the number of new deaths.

After the numerical data are added, the equation 2 converts to *Y*=*X*_1_**·**0.017+ +*X*_2_**·**0.01–13.31.

Following this step, the coefficient of determination *R* increased to 0.8 thus indicating an even greater accuracy.

For example, at the peak of the epidemic in China, as of February 4, 2020, 64 deaths were recorded, and as of February 5, 2020, another 66 deaths were added. Substituting these numbers in the above model, we get: *Y*=3235 infected cases×0.017+2788 severe cases×0.01–13.31=69 deaths. This result is very close to the actual mortality observed on February 4–February 5, 2020.

In addition to improving the predictive ability, an important advantage of the expanded model (model 2) is the absence of a time lag between the prediction and the real outcome date; this advantage should be considered in developing of improved models.

## Conclusion

The developed regression models for predicting the spread of infection and the number of deaths can be used in the current epidemiological situation in Russia. Here, there is a rapid change in primary data coming from different sources followed by clarification and correction of this information. The initial model presented in this study can serve for an approximate assessment of the situation, and the expanded model can increase the accuracy of prediction and improve the analytic algorithm.
